# Monocytes and neutrophils in the inflammatory cascade of systemic onset Juvenile Idiopathic Arthritis

**DOI:** 10.1186/1546-0096-13-S1-O54

**Published:** 2015-09-28

**Authors:** N ter Haar, W de Jager, R Scholman, P Leliefeld, T Tak, B Vastert, S de Roock

**Affiliations:** 1 University Medical Center Utrecht, Laboratory for Translational Immunology, Utrecht, Netherlands; 2 University Medical Center Utrecht, Department of Pediatric Immunology, Utrecht, Netherlands

## Background

Systemic onset Juvenile Idiopathic Artritis (sJIA), also known as Still's disease, is characterized by arthritis with symptoms of systemic inflammation such as spiking fever, rash and serositis. It is considered an autoinflammatory disease with a major role for the innate immune system, reflected by extremely high serum levels of S100 proteins and interleukin (IL)-18. How the number of monocytes and neutrophils relate to the increased levels of S100-proteins and IL-18 and to sJIA disease progression is still unknown.

## Objective

To study the role of monocytes and neutrophils in the inflammatory cascade of sJIA.

## Methods

We determined *ex vivo* cell frequencies and cell surface activation markers of sJIA patients at disease onset, in remission and healthy controls by flow cytometric analysis. For the *in vitro* assessment of neutrophils, we stimulated whole lysed blood or isolated neutrophils with S100-proteins, IL-18, platelet-activating factor (PAF) with or without Formyl-Methionyl-Leucyl-Phenylalanine (fMLP) or phorbol 12-myristate 13-acetate (PMA) and determined intracellular ROS production, degranulation and apoptosis. To investigate the role of monocytes, we stimulated peripheral blood mononuclear cells (PBMCs) from sJIA patients and healthy controls with S100-proteins (+/- ATP) or other TLR-ligands and determined the concentration of cytokines in the supernatant by multiplex immunoassays.

## Results

Patients with new onset sJIA had significantly elevated neutrophil counts compared to healthy controls and sJIA patients with clinically inactive disease, while the amount of monocytes was not significantly different between the groups. Neutrophils from new onset sJIA patients showed an activated phenotype, reflected by higher *ex vivo* expression of Fc-gamma receptors (CD32 and CD64), markers of secretory vesicles (CD35) and specific granules (CD66b) compared to healthy controls. Neutrophils from new onset sJIA showed enhanced ROS production and degranulation and appeared to be more resistant to apoptosis. In contrast to the hyperactivated status of neutrophils in active sJIA,, PBMCs from these patients produced less IL-18 upon S100 stimulation compared to PBMCs from the same patient in remission or healthy controls. The same trend was observed when PBMCs from sJIA patients were stimulated with LPS, TLR2- or TLR7/8 ligands, suggesting cross-tolerance in these patient cells.

## Conclusions

Although monocytes from sJIA patients with active disease are less responsive towards stimulation, neutrophil counts, ROS production and degranulation are clearly elevated. The exact role of each cell type and activity and their interaction in sJIA pathology is currently under investigation.

